# Gut Microbiota Is Not Essential for Survival and Development in *Blattella germanica*, but Affects Uric Acid Storage

**DOI:** 10.3390/life14010153

**Published:** 2024-01-21

**Authors:** Rebeca Domínguez-Santos, Joaquín Baixeras, Andrés Moya, Amparo Latorre, Rosario Gil, Carlos García-Ferris

**Affiliations:** 1 Institute for Integrative Systems Biology (I2SysBio), Universitat de València/CSIC, Calle Catedrático Agustín Escardino, 9, 46980 Paterna, Spain; rebeca.dominguez@uv.es (R.D.-S.); andres.moya@uv.es (A.M.); amparo.latorre@uv.es (A.L.); 2 Genomic and Health Area, Foundation for the Promotion of Sanitary and Biomedical Research of the Valencia Region (FISABIO), Avenida de Cataluña, 21, 46020 Valencia, Spain; 3 Cavanilles Institute of Biodiversity and Evolutionary Biology (ICBiBE), University of Valencia, Calle Catedrático José Beltrán, 2, 46980 Paterna, Spain; joaquin.baixeras@uv.es; 4 Departament de Bioquímica i Biologia Molecular, Universitat de València, Calle Dr. Moliner, 50, 46100 Valencia, Spain

**Keywords:** *Blattella germanica*, germ-free, *Blattabacterium*, gut microbiome, fitness, uric acid reservoir

## Abstract

Cockroaches harbor two coexisting symbiotic systems: the obligate endosymbiont *Blattabacterium cuenotii*, and a complex gut microbiota. *Blattabacterium* is the only bacterium present in the eggs, as the gut microbiota is acquired by horizontal transmission after hatching, mostly through coprophagy. *Blattella germanica*, a cosmopolitan omnivorous cockroach living in intimate association with humans, is an appropriate model system for studying whether the gut microbiota is essential for the cockroach’s survival, development, or welfare. We obtained a germ-free cockroach population (i.e., containing normal amounts of the endosymbiont, but free of microbes on the insects’ surface and digestive tract). Non-significant differences with the controls were detected in most fitness parameters analyzed, except for a slight shortening in the hatching time of the second generation and a reduction in female weight at 10 days after adult ecdysis. The latter is accompanied by a decrease in uric acid reserves. This starvation-like phenotype of germ-free *B. germanica* suggests that the microbiota is not essential in this species for survival and development throughout its complete life cycle, but it could participate in complementation of host nutrition by helping with food digestion and nutrient absorption.

## 1. Introduction

Mutualistic stable symbioses with bacteria have evolved independently many times in all eukaryotic lineages, allowing them to occupy a diversity of ecological niches. Consequently, this phenomenon has had a significant impact on animal evolution [1,2,3]. Insects are the most diverse group and with more species of all terrestrial animals [4], being an essential component of ecosystems and affecting many aspects of human life. Many of them live in obligate relationships with different symbiotic bacteria, which are needed to maintain their hosts’ fitness in their natural environment [5,6]. Cockroaches (members of order Blattodea together with termites) are considered one of the animal groups with the greatest adaptive capacity and evolutionary success, as they are able to exploit constantly changing environments [7]. They are an interesting model because they harbor two symbiotic systems that coexist in the same organism. One of them is the obligatory (primary) endosymbiont *Blattabacterium cuenoti* [8] (hereinafter *Blattabacterium*), a bacterium belonging to the class Flavobacteria in the phylum Bacteroidota, which lives inside specialized cells called bacteriocytes, located in the fat body tissue; in addition, they also harbor a rich and complex gut microbiota [9], mostly located in the posterior part (hindgut) of the digestive tract [10]. 

The endosymbiont is transovarially transmitted from mothers to their offspring. During the nymphal stage, a few bacteriocytes migrate to the ovaries, where the endosymbionts are released. Once in adult stage, they become incorporated into developing oocytes after vitellogenesis and before chorionization and ovulation [11,12,13]. For its part, the gut microbiome is horizontally transmitted, mostly through the intake of congeners’ feces from the environment (coprophagy) [14,15,16]. Thus, in the German cockroach *Blattella germanica* (Blaberoidea:Ectobiidae), the species used in this work, it has been demonstrated that *Blattabacterium* is the only bacterium present in the eggs, and the microbiota is definitely established in the second nymphal instar, acquired from the environment, mainly from the feces of the population [17]. 

Genome sequencing, transcriptomics and metabolic analyses have demonstrated that *Blattabacterium* has an essential role in host nutrition, even though cockroaches are omnivorous insects. Similar to other insects’ obligate endosymbionts, it provides the host with nutrients, such as essential and some non-essential amino acids, as well as some vitamins. Additionally, it is an essential participant in nitrogen recycling through the use of uric acid deposits accumulated in other fat body-specialized cells, the urocytes, allowing the insect to take advantage of a frequent excess of protein nitrogen in its diet [18,19,20,21,22]. Specifically, the uric acid is transformed by the host through the uricolytic pathway into urea, which enters *Blattabacterium* to be degraded into CO_2_ and ammonia by the endosymbiotic urease; then, the ammonia can be fixed into glutamate and glutamine by the endosymbiont and the host, respectively. Regarding the gut microbiota, most studies on cockroaches have focused on microbiota composition, assembly during developmental stages, and putative roles in host physiology [15,17,23,24,25,26,27]; however, the empirical determination of its functions and effect on insect fitness need to be addressed. Furthermore, it would be interesting to know if the gut microbial community can somehow replace the endosymbiont’s essential functions. To do so, two opposite and complementary approaches can be followed, eliminating *Blattabacterium* without affecting the gut microbiota and eliminating the gut microbiota without affecting *Blattabacterium*. In previous studies performed in our laboratory [15,28], *B. germanica* populations were treated with rifampicin during the oocyte infection stage, to drastically reduce the endosymbiont population in the next generation, obtaining quasi-aposymbiotic individuals. It is worth mentioning that fully aposymbiotic populations could not be obtained because they were unable to reproduce, due to a lack of *Blattabacterium*. We found that, after antibiotic treatment, while the endosymbiont population remained extremely reduced in G2 and the microbiota was able to recover, the latter could not compensate for the endosymbiont role, as host fitness was drastically affected [28]. However, the effect on fitness of a total absence of gut microbiota while keeping a normal endosymbiont load, remained to be determined. To do this, it was necessary to maintain the population in a germ-free environment. 

Production of germ-free animals is useful for helping to understand the role played by the different components of the natural microbiota. For this reason, the axenic culture of invertebrate metazoa has been the focus of many studies since the beginning of the twentieth century [29]. Different chemical reagents have been used to sterilize eggs without killing the embryos, to obtain germ-free insects [23,30,31,32,33,34]. As for cockroaches, different results have been observed regarding the microbiota’s effect on maturation and reproduction of individuals, depending on the species under study. Thus, germ-free *Periplaneta americana* (Blattoidea:Blattidae) displayed prolonged growth rates and gut tissue dysmorphias compared with control insects [27], and underwent notable developmental and morphological changes [35]. However, most studies on *B. germanica* show about the same generation time in control and axenic populations [14,32,36], and there are no additional reports on the effect of lack of gut microbiota on other developmental and fitness parameters in this species. 

In the present work, taking profit from the previous knowledge regarding gut microbiota transmission, we implement a *B. germanica* germ-free population in which we imped the establishment of the microbiome while maintaining the primary endosymbiont, and we determine different host fitness parameters. Even though our experiments show that axenic rearing conditions impact some aspects of *B. germanica* growth, such as adult female weight and uric acid storage, most survival and developmental parameters remain unchanged, and the cockroaches can reach adult stage and reproduce in these sterile conditions. This will allow us to design new experiments by exposing the cockroach’s germ-free populations to different challenging conditions to delve into the role of the gut microbiota prior to understanding if there is a crosstalk between the two spatially separated symbiotic systems.

## 2. Materials and Methods

### 2.1. *Blattella germanica* Rearing Conditions

Cockroaches were reared in plastic or glass jars with aeration inside climatic chambers (Inkoa Ca00/15, Erandio, Spain) at the I2SysBio (University of Valencia-CSIC), at 25 °C, 60% relative humidity, and 12 h light/12 h darkness photoperiod (cool white fluorescent tubes, irradiance around 20 W/m^2^ on the containers). Insects were fed with dog food pellets (Teklad global 21% protein dog diet 2021C, Envigo, Madison, WI, USA) and water ad libitum. When needed, dog food pellets were autoclaved at 121 °C for 20 min.

### 2.2. Generation of Germ-Free Cockroaches and Quality Control

Adult cockroaches were collected between 0 and 48 h after adult ecdysis to create a synchronized sex-balanced population. Mature oothecae were isolated from females when the embryo was fully developed (i.e., the dark macula lying against the ootheca’s ventral surface migrated to the lateral surface, and the green yolk line became clearly visible [30]). The oothecae surface was sterilized following the protocol for *Shelfordella lateralis* with modifications [23]. Briefly, oothecae were immersed for 20 s in 0.1% SDS followed by 12 min immersion in 2% peracetic acid with gentle shaking. Then, they were rinsed twice with sterile water, and transferred to a sterile gauze to remove excess water. Oothecae were placed individually in sterile 50 mL tubes, with sterile tap water and autoclaved food. The tubes were incubated at 25 °C until the hatching and manipulated in a laminar-flow workbench under aseptic conditions. 

In order to evaluate the effectiveness of the sterilization protocol, we placed at least one nymph of each hatching ootheca on the surface of BHI (brain heart infusion) agar plates to detect microbial (bacterial or fungal) growth. The BHI plates were incubated at 37 °C for 24 h, and then transferred to 25 °C for at least four weeks. Furthermore, to check for potential microbial contamination not able to grow in these conditions, DNA extraction from individual nymphs was performed, followed by amplification and sequencing of the V3–V4 region of the 16S rRNA gene. Each whole nymph was ground with a sterile plastic pestle, and total DNA was obtained with the JetFlex Genomic DNA Extraction kit (Genomed, Leinfelden-Echterdingen, Germany), following the protocol previously described [16]. Illumina sequencing was performed at the Sequencing and Bioinformatics Service of FISABIO. Additionally, scanning electron microscopy observations were carried out to check for the presence or absence of gut microbes (see the Section 2.5). 

### 2.3. Experimental Design and Fitness Parameters’ Determination

After hatching, and once their axenic state was confirmed, the nymphs from each sterilized ootheca were used to start a new population in one of three conditions: control fed on sterilized (C-s, *n* = 6), non-sterilized food (C-ns, *n* = 6), or kept in germ-free conditions (GF, *n* = 7). The control populations were raised in a climatic chamber in the above-mentioned conditions, and feces from the laboratory population were added during the first 10 days to allow the acquisition of the parental microbiome. The germ-free populations were raised in aseptic conditions inside autoclaved glass bottles with all sterile materials, in a different insect chamber, and the glass bottles were opened in a laminar-flow workbench every other day to allow air exchange. The axenic state of habitats and individuals in the germ-free populations was checked weekly throughout the experimental phase by plating feces onto BHI plates. Batches with bacterial or fungal growth were discarded.

The following fitness parameters were determined: time from nymph to adult (from ootheca hatching to the first adult ecdysis), weight of adult individuals collected between 0 and 48 h after ecdysis, weight of 10-day-old adult females, time of first ootheca emergence, and time of first ootheca hatching in each population. The last two parameters were assessed from the appearance of the first adult couple. Data were statistically analyzed through a *t*-test to determine whether there was a significant difference when comparing two independent samples, and using an ANOVA test followed by a Bonferroni correction, or a Kruskal–Wallis test followed by a Dunn test, for pairwise comparisons to compare three independent samples.

### 2.4. Cockroach Dissection and In Vivo Visualization of Fat Body Morphology

Ten-day-old adult female cockroaches were dissected under an Olympus SZ61 stereo microscope. Individuals were anesthetized using a CO_2_ stream, and cleaned with different solutions: bleach 10%, ethanol 70%, and two passages in the type II sterile water. Then, they were fixed with pins in a dorsal position on a silicone plate. They were ventrally opened via a longitudinal incision in the abdomen, and hindguts and fat bodies were extracted and gently washed with Krebs–Ringer Bicarbonate Buffer (Sigma-Aldrich, Burlington, MA, USA). Hindguts were immediately fixed for electron microscopy, and fat body samples were frozen in liquid nitrogen and stored at −80 °C until use for DNA extraction and acid uric determination. For in vivo visualization, ventrally-opened individuals were photographed using a Leica Z16 microscope equipped with a CF500 camera and LAS 4.9 (Leica, Wetzlar, Germany) software. Z-stacks, followed by extended depth of field application, were extensively used to produce final images.

### 2.5. Electron Microscopy

Combined techniques of scanning electron microscopy (SEM) and transmission electron microscopy (TEM) were used to visualize the luminal surface of the hindgut of adult *B. germanica* individuals from control and germ-free populations.

Hindguts for SEM were longitudinally cut to expose the internal vestiture, fixed by immersion in Karnovsky’s fixative [37] containing 0.1% Triton X-100 to improve tissues wettability, washed in distilled water, and progressively dehydrated through a graded ethanol series, from 70% to absolute ethanol. Gut pieces were individually placed inside microporous specimen capsules (30 μm pore size) immersed in absolute ethanol, followed by critical point drying in a Leica EM CPD300. The obtained fragments were arranged on SEM aluminum stubs using carbon tape and coated with Au/Pd sputtered in argon gas. Observation and photography were performed in a scanning electron microscope Hitachi S-4800 at the Central Service of Support to Experimental Research (SCSIE) of the University of Valencia.

In the case of TEM samples, the hindgut lumen was cleaned from fecal traces by injecting Ringer solution using a hypodermic syringe. Fragments were fixed for 24 h in 2% glutaraldehyde in Millonig’s phosphate buffer [38] containing 0.54% glucose and 0.005% calcium chloride, and then washed in the same buffer. Fragments so obtained were post-fixed with 2% osmium tetroxide, rinsed, dehydrated, and embedded in Durcupan resin (Fluka). Semithin sections (1.5 µm) were cut with a diamond knife on a Leica EM UC6 ultratome, and lightly stained with 1% toluidine blue for inspection. Ultrathin (0.08 µm) sections were cut with a diamond knife from selected semithin sections, stained with lead citrate (Reynolds solution), and examined and photographed under a transmission electron microscope JEOL JEM-1010.

### 2.6. Fat Body DNA Extraction and *Blattabacterium* Quantification

Total DNA was extracted from fat body following the protocol previously described [16]. DNA was quantified with Qubit 2.0 Fluorometer (Thermo Fisher, Waltham, MA, USA), and used for *Blattabacterium* quantification by qPCR with ArialMx Real-Time PCR System (Agilent Technologies, Santa Clara, CA, USA), using the *ureC* gene (accession number NC_013454.1). The eukaryotic gene *actin5C* (accession number AJ861721.1) was used as an internal control. Specific primer pairs were used for amplifying both genes as previously described [15]. Statistical analysis was performed using a Mann–Whitney test.

### 2.7. Uric Acid Extraction and Quantification

Fat body samples were dried for 72 h at 80 °C and homogenized in 0.1 M lithium carbonate. Uric acid solubilization was performed overnight at 60 °C and 800 rpm, and the supernatant was collected. A dilution of 1 mg (dry weight)/mL was prepared and processed with Amplex Red Uric Acid/Uricase Assay kit (Thermo Fisher Scientific, Carlsbad, CA, USA) according to the manufacturer’s protocol. Quantification of the fluorescence intensity was performed with a Varioskan Lux Reader (Thermo Fisher, Waltham, MA, USA) at wavelength 530 and 590 nm for excitation and emission, respectively. Statistical analysis was performed using a Mann–Whitney test.

## 3. Results and Discussion

The study of host–microbiota interactions greatly benefited from the production and rearing of axenic animals [39]. Many protocols have been used over time to obtain germ-free insects, including cockroaches, with some based on the use of sodium hypochlorite [32,33] and others on the use of peracetic acid [23,30,40]. We tried several approaches using different concentrations of sodium hypochlorite at different times (0.25% for 20 min, 1% for 5 min, and 3.7% for 30 s; the latter treatment also included 0.1% SDS). We had to discard these protocols because the sterilization procedures were too long and some oothecae opened before the end of the protocol. We also tested several peracetic acid-based protocols since these are highly effective disinfectants against all kinds of microbes, including bacteria, filamentous and yeast fungi, and even spores and viruses. We tested different concentrations of peracetic acid (0.1 or 2%), treatment times (5, 10, 12, 15 and 20 min), and durations of previous immersions in 0.1% SDS (5, 20, 30 and 60 s). With the protocol used in this work, based on [23] with modifications (immersion for 20 s in 0.1% SDS followed by 12 min immersion in 2% peracetic acid with gentle shaking), we obtained the best results in terms of sterility, number of oothecae that hatch after the sterilization process, and number of viable nymphs per ootheca.

**Figure 1:**
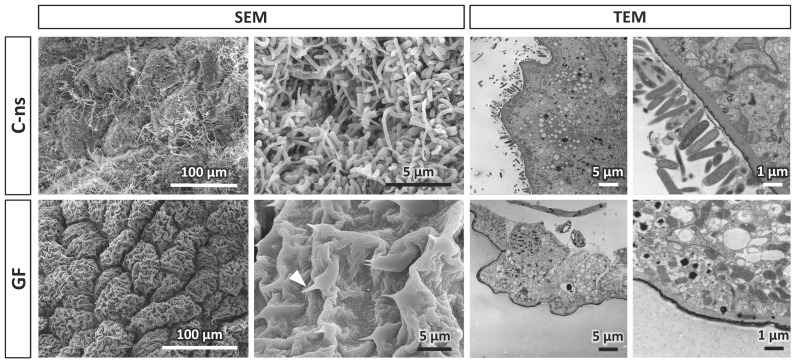
Comparison of hindgut morphology in 10-day-old adult females of *B. germanica*. Scanning electron microscopy (SEM) images of the surface covering the lumen of the hindgut and transmission electron microscopy (TEM) images of a transversal section of the hindgut of control (feed with non-sterilized food, C-ns) and germ-free (GF) specimens. The left and right panels show different image magnifications, as indicated in the images. The arrowhead points to a cuticular microprotuberance.

In addition to the microbiological verification of the axenic state of the germ-free samples, we performed SEM and TEM analyses (Figure 1). In control individuals, SEM images revealed the presence of different bacterial shapes on the hindgut surface, mostly cocci and bacilli. The same intestinal wall appeared completely clean in germ-free cockroaches. In fact, cuticular microprotuberances (acanthae) that avoided the retrogression of digested food, while the chyme advances through the gut were clearly visible as the epithelial cells were not covered by the microbial biofilm. This was correlated with TEM inspection of transversal sections of the hindgut, where most gut microbiota is located in cockroaches. In control samples, a stacking of bacterial cells was clearly observable on the intestinal wall, while no bacterial arrangement was detectable in germ-free hindguts.

**Figure 2:**
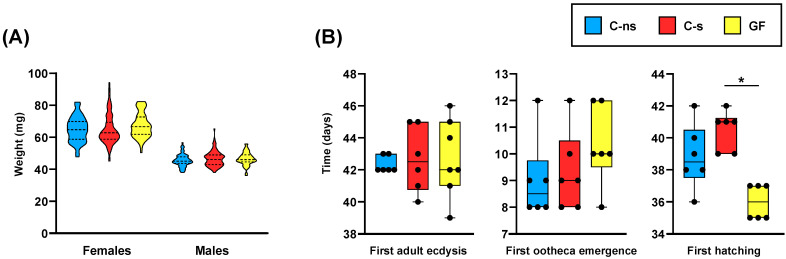
(**A**) Violin plots of adult weight at 0–48 h after ecdysis. Statistical analysis performed using an ANOVA test with a Bonferroni correction. (**B**) Box plots showing different development time parameters in each population: the earliest adult ecdysis (**left**), the earliest emergence of an ootheca (**center**), and the first hatching (**right**). The two later parameters were measured from the establishment of the first couple of individuals. Statistical analysis was performed using a Kruskal–Wallis test followed by a Dunn test. The asterisk bar indicates where significant differences between groups were detected (C-s vs. GF, *p*-value = 0.0029). Abbreviations: C-ns, control specimens fed with non-sterilized dog food; C-s, control specimens fed with sterilized dog food; GF, germ-free specimens.

Because we needed to feed the germ-free population with autoclaved food, which could affect the nutritional value of the diet by destroying thermolabile components, we first compared the fitness of control populations fed with sterilized (C-s) or non-sterilized (C-ns) dog food. We did not observe significant differences between these two control populations. In fact, the time window in which adult ecdysis occurred overlapped between these two control populations (Table 1). Furthermore, the weight of adult individuals 0–48 h after ecdysis (Figure 2A and Table S1), the time needed to reach the first adult ecdysis, the time needed for the emergence of the first ootheca, and the time until the first hatching in each population (Figure 2B) were similar in both cases. Consequently, if some degradation of thermolabile nutrients occurred during food sterilization, it did not affect these fitness parameters.

Regarding the developmental fitness parameters, we did not observe any delay in the germ-free (GF) population in reaching the adult stage, the time required for the first adult ecdysis since hatching, or the time between the appearance of the first adult couple and the emergence of the first ootheca, compared to both control conditions (Figure 2B). We only observed significant differences in the time that elapsed from the emergence of the first couple and the first ootheca hatching in G2 (Figure 2B), slightly shorter in germ-free cockroaches compared to the C-s populations (*p*-value = 0.0029). Thus, under our working conditions, the microbiomes normally present in the control populations did not seem to contribute significantly to cockroach development. Although some pioneering works on the generation of germ-free *B. germanica* populations reported slightly longer maturation times compared to control populations [36], our results match other work in which both germ-free and control populations share approximately the same generation time [14,32]. This observation would be compatible with the possibility that the ootheca counted as first hatched in C-s and C-ns did not correspond to the first emerged ootheca, but to another that could have emerged a few days later, the first one having fallen. If so, this would mean that there were no differences between control and germ-free populations on this fitness parameter. It is worth mentioning that in previous experiments carried out in our laboratory with populations under control conditions (equivalents to C-ns), the hatching time counted from the emergence of the first ootheca was similar to that of the GF population in this experiment. Additional experiments should be performed to test this hypothesis. 

**Figure 3:**
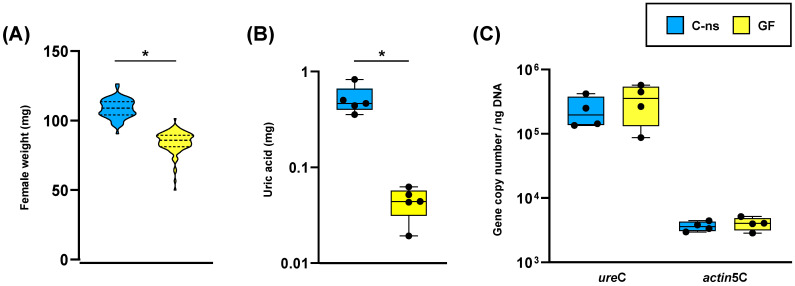
(**A**) Weight of 10-day-old adult females. (**B**) Weight of uric acid stored in the fat body. (**C**) Endosymbiont load in the fat body measured with qPCR of the *Blattabacteriun* gene *ureC*; the host gene *actin5C* has been used as an internal control. Statistical analyses were performed using a *t* test (panel **A**) or a Mann–Whitney test (panels **B**,**C**). The asterisk bar indicates where significant differences between groups were detected (panel **A**, *p*-value < 0.0001; panel **B**, *p*-value = 0.0079). Abbreviations: C-s, control specimens fed with sterilized dog food; GF, germ-free specimens.

**Figure 4:**
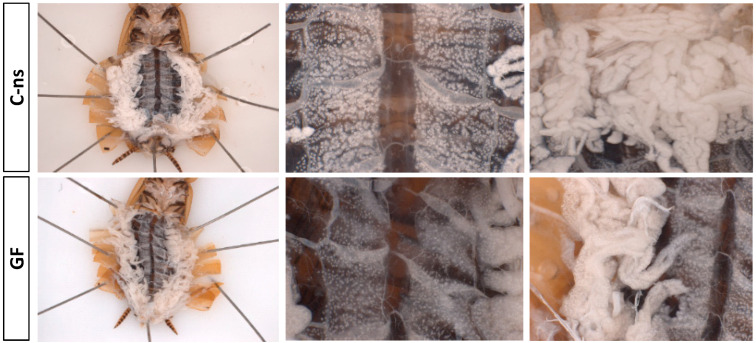
Comparison of the fat bodies from control and germ-free 10-day-old adult females. In vivo visualization of the fat body morphology (**left**) and details of the peripheral (**center**) and visceral (**right**) regions. Abbreviations: C-ns, control specimens fed with non-sterilized dog food; GF, germ-free specimens.

Similar fitness studies have been performed on germ-free populations of *P. americana* [27,35] with a very different outcome, as these cockroaches exhibit notable developmental and growth defects, such as prolonged development time, reductions in body size, and gut tissue dysmorphias. In our experiment, when we compared germ-free cockroaches to both control populations regarding the weight of adult individuals 0–48 h after the ecdysis (Figure 2A and Table S1), we found no significant differences between both sexes. However, there were significant differences in female weight at 10 days after adult ecdysis, with lower weight in the germ-free condition (Figure 3A). Interestingly, when dissecting 10-day-old females, we observed that germ-free ones had a slightly clearer fat body (Figure 4), which correlated with a drastic reduction in uric acid deposits (Figure 3B). Because uric acid is the way to store the surplus of nitrogen from the diet, it might indicate that the absence of gut microbiota was negatively affecting the capability to digest and absorb nutrients. This is similar to the starvation-like phenotype that has been described in germ-free *P. americana* [27,35], and could explain the observed lower weight of 10-day-old germ-free females in our experiments. In addition, and as expected, the quantification of *ureC* demonstrated that the endosymbiont load was not affected by the lack of microbiota (Figure 3C). Therefore, *Blattabacterium* is not recycled in this situation, as was described for *P. americana* under starvation conditions [41], because it is necessary for the production of essential amino acids and some vitamins [18,19,22] and will be able to process urea, obtained from the mobilized uric acid, whenever the conditions become normal.

## 4. Conclusions

The microbiota is not essential for *B. germanica* survival, development, and reproduction under sterile conditions. However, the ability to store nutrients is negatively affected by its absence, probably because the lack of microbiota has a negative impact on the digestion of food and the availability of nutrients for absorption. Nevertheless, germ-free populations can be maintained over generations because *Blattabacterium* is still present in normal amounts, and survival is not affected. Consequently, experimental studies of controlled microbiota implantation under challenging conditions are possible. This methodology supports measuring the evolutionary success of cockroaches in different environments as well as the differential contribution of each of the symbiotic systems.

## Figures and Tables

**Table 1:** Number of individuals that reached adult ecdysis over time in the different conditions under study. Abbreviations: C-ns, control specimens fed with non-sterilized dog food; C-s, control specimens fed with sterilized dog food; GF, germ-free specimens.

Condition	Number of Oothecae	Number of Individuals Reaching Adult Ecdysis Each Day (d)															Total Individuals
		40 d	42 d	44 d	46 d	48 d	50 d	52 d	54 d	56 d	58 d	60 d	62 d	64 d	66 d	68 d	
C-ns	6	0	10	35	29	12	16	14	8	3	3	0	0	0	0	0	130
C-s	6	0	22	37	28	15	28	29	16	9	2	1	2	0	0	1	190
GF	7	4	7	9	27	17	26	22	6	0	0	0	0	0	0	0	118

## Data Availability

The data presented in this study are available on request from the corresponding author.

## Supplementary Materials

The following supporting information can be downloaded at: https://www.mdpi.com/article/10.3390/life14010153/s1, Table S1. Weight of adults 0–48 h after adult ecdysis in all studied conditions.
